# Complex response to physiological and drug-induced hepatic heme demand in monoallelic *ALAS1* mice

**DOI:** 10.1016/j.ymgmr.2021.100818

**Published:** 2021-11-12

**Authors:** Viktoria Vagany, Susan Robinson, Tatyana Chernova, Andrew G. Smith

**Affiliations:** a Medical Research Council Toxicology Unit, University of Cambridge, United Kingdom; b Department of Genetics and Genome Biology, University of Leicester, United Kingdom

**Keywords:** Aminolevulinic acid synthase 1, Semi null mice, Hepatic response, Compensation, complex regulation, ALAS1, aminolevulinate synthase 1, AIP, acute intermittent porphyria, FECH, ferrochetalase, HMOX1, heme oxygenase 1, PCR, polymerase chain reaction, WT, wild type, 4-ethyl-DDC, 4-ethyl-3,5-diethoxycarbonyl-2,6-dimethyl-1,4-dihydropyridine

## Abstract

Regulation of 5-aminolevulinate synthase 1 (ALAS1) for nonerythroid heme is critical for respiration, cell signaling mechanisms and steroid/drug metabolism. ALAS1 is induced in some genetic disorders but unlike other genes in the heme pathway, a gene variant of *ALAS1* associated with inherited disease has not been reported. BALB/c mice carrying a null *ALAS1* allele caused by a *βGEO* insert were developed and used to determine the consequences of heme demand of a semi gene copy number. Homozygous disruption of *ALAS1* (−/−) was lethal for embryo development post day 6.5 but expression in heterozygotes (+/−) was sufficient for the number of offspring and survival. In both wild type (WT +/+) and +/− mice expression of ALAS1 RNA was greatest in liver and harderian gland and much lower in kidney, lung, heart, brain and spleen. The effects of one WT *ALAS1* allele in +/− mice on mRNA levels in liver and harderian gland were less marked compared to brain and other organs that were examined. Many other genes were up-regulated by heterozygosity in liver and brain but to a minimal extent. Hepatic heme oxygenase 1 (HMOX1) mRNA expression was significantly lower in +/− mice but not in brain. No elevated translation of WT allele ALAS1 mRNA was detected in +/− liver as a compensatory mechanism for the disabled allele. Fasting induced ALAS1 mRNA in both WT and +/− mice but only in +/− was this manifest as increased ALAS1 protein. The hepatic protoporphyria-inducing drug 4-ethyl-DDC caused induction of hepatic ALAS1 mRNA and protein levels in both WT and +/− mice but markedly less in the mice with only one intact allele. The findings illustrate the complex response of *ALAS1* expression for heme demand but limited evidence that upregulation of a wild type allele can compensate for a null allele.

## 1 Introduction

**Fig. 1:**
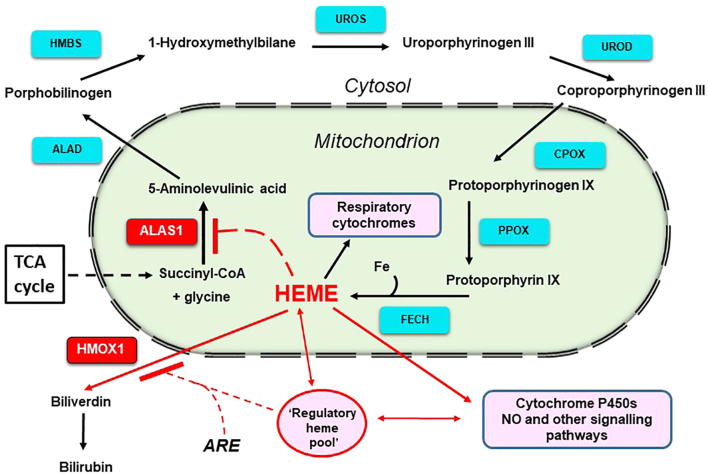
Nonerythropoietic metabolism of heme. Heme is synthesized from glycine and succinyl-CoA, through eight enzymatic steps that take place in the cytoplasm and the mitochondrion. Heme negatively regulates its own synthesis via targeting the first and rate-liming enzymatic step and is metabolized to bilirubin if in excess for requirements or as an antioxidant response under stress. Synthesis is probably tightly linked to mitochondrial function and the TCA cycle depending on physiological requirements. ALAS1, 5-aminolevulinic acid synthase; ALAD, ALA dehydratase; HMBS, 1-hydroxymethylbilane synthase; UROS, uroporphyrinogen III synthase; UROD, uroporphyrinogen decarboxylase; CPOX, coproporphyrinogen oxidase; PPOX, protoporphyrinogen IX oxidase; FECH, ferrochelatase; HMOX1, heme oxygenase 1; ARE, antioxidant response element.

Nonerythroid heme supply is fundamental for cellular function including mitochondrial respiration, pharmacologically pertinent systems such as cytochromes in drug and steroid metabolism, the synthesis and operation of NO and microRNAs, and the signaling of some ion channels [1]. Inherited disorders of heme synthesis (porphyrias) and triggers have been studied widely [2]. The first specific precursor, 5-aminolevulinate (5-ALA), is formed from succinyl CoA and glycine in mitochondria by 5-aminolaevulinic acid synthase 1 (ALAS1) (Fig. 1). Regulation of expression of *ALAS1* plays a key role, in part by feedback control of a regulatory heme pool, and may vary with cell type and physiological demands as well as being modulated post-transcriptionally. In addition, endogenous factors such as steroids and bile acids and exogenous chemicals such as some drugs and environmental agents can markedly stimulate expression [3], [4]. Erythropoietic formation of 5-ALA is dependent on the *ALAS2* gene regulated by iron supply [5]. Heme superfluous to requirements is metabolized by inducible microsomal heme oxygenase 1 (HMOX1) to bilivirdin, CO and iron [1], [3]. Homeostasis of heme formation and export seem to be inter-dependent with mitochondrial functioning and thus has important physiological consequences [6]. In addition, when the TCA cycle is restricted at fumarate hydratase, due to mutations in some tumors and in a model of human cardiovascular disease, the pathway from 5-ALA to heme oxidation is co-opted into a route for disposal of succinyl CoA formed from glutamine as a source of generating NADH [7], [8].

Many studies of *ALAS1* expression have focused on hepatic up-regulation especially in acute human porphyrias, particularly acute intermittent porphyria (AIP), caused by autosomal dominant mutations in down-stream enzymes of heme synthesis and triggered by hormones, drugs, alcohol, and other chemicals, or by nutritional demands [1], [2]. In these circumstances, compensatory up-regulation of hepatic *ALAS1* may lead to toxic neurovisceral levels of 5-ALA. Similar findings occur in type 1 tyrosinemia and lead poisoning [9]. Importantly, whereas known inherited disorders of heme synthesis are the consequence of dominant or recessive variants of the other genes of the pathway no human porphyrias or other clinical conditions have been associated with *ALAS1* [2]. This could be explained by any variant of *ALAS1* that arises is lethal in embryo development and thus is not observed in children or adults. Alternatively, a variant may give rise to an enzyme protein that is less efficient but is sufficiently active for most cellular demands. Finally, feedback up-regulation or other modulations may compensate for deficiency in enzyme activity [10].

In mice there is little evidence of compensatory regulation of the genes of the heme synthetic pathway (Fig. 1) in response to mutations or gene dosage. Strain variations in expression of 5-aminolevulinate dehydratase are reflections of gene copy number [11], [12], [13]. In knockout mice heterozygosity for 1-hydroxymethylbilane synthase or uroporphyrinogen decarboxylase genes leads to hepatic mRNA levels that are approximately 50% of homozygous, and in combination with external challenges are associated with pathological outcomes [14], [15]. Exon deletion or mutation of the ferrochelatase (*FECH*) gene are not associated with compensatory upregulation of hepatic FECH mRNA despite marked pathological phenotypes [16], [17], [18]. Basal levels of ALAS1 mRNA in mice with a *GFP* gene insert in one *ALAS1* allele on a mixed genetic background were reported as 50% of the homozygous animals [19].

However, unlike other genes in the heme synthetic pathway, hepatic ALAS1 expression is highly sensitive to physiological changes and stimulation by drugs or chemical-induced pathogenicity which might compensate for effects on normal gene dosage at transcriptional, posttranslational and protein maturation and degradation levels [1], [20]. Basal levels of hepatic ALAS1 activity and mRNA in mice can also be genetically determined without apparent phenotypic consequences [12], [21]. Hence *ALAS1* expression might be complicated by a complex genetic and physiological landscape.

An *ALAS1* null mouse line disabled by a β-galactosidase insert was bred onto a single genetic background [22]. Adult heterozygotes were studied to explore the influence of one null functioning *ALAS1* allele compared to homozygotes (WT) on survival and the expression of ALAS1 under basal states and to examine evidence for compensatory regulation after physiological and pharmacological challenge.

## 2 Materials and methods

### 2.1 Chemicals

Phenobarbital was obtained from Sigma-Aldrich (Dorset, UK) and 4-ethyl-3,5-diethoxycarbonyl-2,6-dimethyl-1,4-dihydropyridine (4-ethyl-DDC) was prepared by A. H. Gibbs [23], [24].

### 2.2 Generation of ALAS1 null mice

**Fig. 2:**
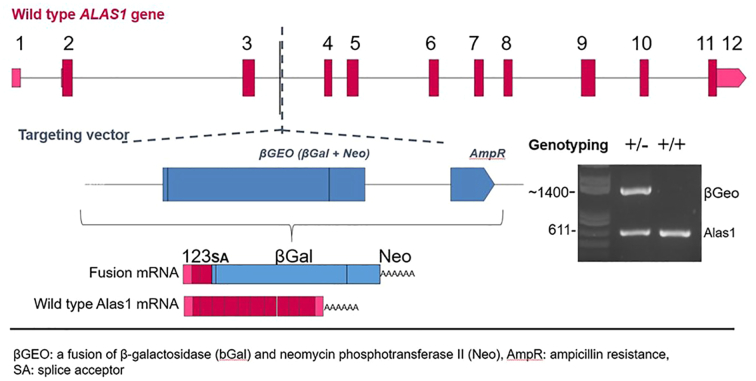
Scheme of β-galactosidase gene inserted into mouse ALAS1 gene by gene trapping approach [22], [25]. Agarose gel shows presence of one βGEO insert in one ALAS1 allele in +/− mice.

A 129/Ola mouse embryonic stem cell line (ID: YTA357) supplied by BayGenomics (San Francisco, USA) was generated that contained a gene-trap vector (*βGEO)* consisting of an upstream fusion of reporter genes β-galactosidase and neomycin phosphotransferase II with AmpR (ampicillin resistance) inserted into an intronic region of the *ALAS1* genomic DNA [22], [25]. Sequencing showed that the resulting insertional mutation created a fusion transcript containing sequences from exons 1–3 of *ALAS1* plus the *βGEO* marker producing a cell line where the vector successfully terminated the transcription of one allele of the *ALAS1* gene between exons 3 and 4 (Fig. 2). Chimeric male mice were bred by injecting the ES cells containing the vector into blastocysts of C57BL/6 J mice under the expert supervision of Dr. S Munson and Geneta colleagues in the University of Leicester. The resulting chimeric mice were backcrossed on to the BALB/c background for 20 generations alternating the sex of the parents.

Mice were also backcrossed on to the C57BL/6J background for 5 generations. The mouse line development, treatments and subsequent expression studies were carried out in the MRC Toxicology Unit when sited in the University of Leicester prior to transfer to the University of Cambridge in 2020. For simplification of comparison between species, abbreviations of genes are shown in uppercase for both mouse and human equivalents.

### 2.3 Treatments

BALB/c and C57BL/6J mice for breeding were obtained from Charles River, UK. All mice were kept on standard bedding in a 12 h–12 h light-dark cycle at 20–22 °C and fed LabDiet 5LF2 diet. Investigations were carried out mostly on young adult mice up to 15 weeks of age. 5-ALA administered to female mice was dissolved in drinking water (1 mg/ml) made fresh every 3 days. The dose administered was approximately 200 mg/kg body weight/day. Animals were killed by cervical dislocation in the morning i.e. 2–6 h in the light period. For fasting studies, diet was withdrawn from mice overnight 18 h before termination of investigations. A single dose of phenobarbital (80 mg/kg) was administered by i.p. injection dissolved in saline. 4-Ethyl-DDC (100 mg/kg) was dosed in vegetable oil (10 ml/kg) [23], [24], [26]. Breeding and in vivo investigations were performed under the U.K. Animals (Scientific Procedures) Act, 1986, Home Office license 60/4529 (Disorders of haem metabolism and links to disease) and University of Leicester ethical requirements.

Protoporphyrin IX was estimated by spectrofluorimetry as previously described except in multiwell plates [24]. Total heme was estimated using the Quantichrome heme assay kit (Bioassay Systems, CA, USA).

### 2.4 Genotyping

Genomic DNA was obtained by lysing tissues using lysis buffer (Tris:Cl pH 8.5100 mM, EDTA 5 mM, SDS 0.2 *w*/*v*%, NaCl 200 mM, 1 μl proteinase K) followed by thermal mixing at 55 °C for 2 h and isopropanol extraction. PCR was performed using Extensior Hi-fidelity PCR Reddymix master mix (ThermoFisher Scientific, Loughborough, UK) and following primers: ALAS1F: 5’-GGAACACCGCAGTAAGTGCT-3′; ALAS1 R: 5’-AACCATGCTGTGCTGGAGTA-3′; β-GEO5: 5’-GACAGTATCGGCCTCAGGAA-3′. Expected products: ~1400 bp knockout and ~ 611 bp wild type.

### 2.5 RNA extraction and quantitative real-time PCR analysis (qPCR)

Tissues were collected and total RNA was isolated by using TRI-reagent (Sigma-Aldrich, Dorset, UK). cDNA synthesis was carried out using random primers and Superscript III (ThermoFisher Scientific, Loughborough, UK). PCR primers were selected using the Primer Express v3.0.1 Software program (ThermoFisher Scientific, Loughborough, UK). Primer sequences are shown in Supplementary Table 1. Primers were designed to cross exon-exon boundaries and the concentration optimized (300–900 nM) to ensure that the efficiency of the target amplification and the efficiency of the endogenous reference amplification (β-actin or β-microglobulin) were approximately equal. PCR was performed using SYBR Green PCR Master Mix, primers and 10 ng of reverse transcribed cDNA in the ABI PRISM 7700 Sequence Detection System (Applied Biosystems, Foster City, CA). The thermal-cycler protocol was: stage one, 50 °C for 2 min; stage two, 95 °C for 10 min; stage three, 40 cycles at 95 °C for 15 s and 60 °C for 1 min. Each sample was run in triplicate. Quantification was performed using the comparative CT method (ΔΔCT). Data presented as the mean ± S.D. (*n* = 3–10 for each group). Statistical significance was assessed as *p* < 0.05 using two-tailed Student's *t-*test.

### 2.6 Immunoblotting

Proteins were extracted from snap-frozen tissues using lysis buffer (0.5% NP-40, 20 mM Tris-HCl, pH 7.5, 137 mM NaCl, 10% glycerol, 2 mM EDTA, 1 mM sodium orthovanadate, 10 μg/μl leupeptin, and 10 μg/μl aprotinin) followed by brief sonication and run on SDS-PAGE (20 mg protein). Proteins were transferred onto nitrocellulose membranes (Bio-Rad laboratories, Hemel Hempstead, UK) using electrophoresis. Membranes were pre-incubated with 5% skimmed milk in TBS-T. After incubation with primary and secondary antibodies, bands were detected by enhanced chemiluminescence (GE Healthcare, Buckinghamshire, UK) and visualized by exposure to X-ray films (Hyperfilm ECL; Amersham Biosciences, UK). Primary antibodies were from the following sources: anti-VDAC1 anti-ALAS1, α-tubulin were from Abcam (Cambridge, UK). Results were quantified using densitometry and Image Quant 5.2 software. Statistical significance of data was estimated using two-tailed student's *t*-test.

### 2.7 β-Galactosidase activity

Sections (10 mm) were cut and mounted on poly-l-lysine-coated slides and allowed to dry for 30 min at room temperature. Staining was done using β-Galactosidase Reporter Gene Staining Kit (Sigma-Aldrich, UK) according to manufacturer instruction. Indigo blue color visualized microscopically indicated expression of reporter gene in tissue sections.

### 2.8 Sucrose density gradient centrifugation and RNA detection

Livers were perfused with 100 μg/ml cycloheximide (Sigma-Aldrich, UK) prior to harvesting. Tissues were lysed in gradient buffer (300 mM NaCl, 15 mM MgCl2, 15 mM Tris pH 7.5, 100 μg/ml cycloheximide) plus 1% Triton X-100. Post nuclear supernatants were layered on 10–50% (*w*/*v*) sucrose gradients dissolved in gradient buffer. Gradients were centrifuged at 38,000 rpm for 2 h at 4 °C in a SW40Ti rotor (Beckman Coulter), then separated through a live optical density (OD) 254 nm UV spectrometer (Isco). Gradients were fractionated with continuous monitoring at 254 nm. RNA was isolated from each fraction using TRIzol reagent (Fisher Scientific, Loughborough, UK). Relative ALAS1 mRNA abundance in the gradient fractions was measured by qPCR. Quantification of β-actin and PABP1 was performed in order to confirm the position of subpolysomes and polysomes.

### 2.9 Transcriptomics

Total RNA from tissues was extracted with TRIzol (Fisher Scientific, Loughborough, UK). Hybridization to 60 K whole mouse genome microarray gene expression chips was conducted following manufacturer's protocol (Agilent Technologies, Berkshire, UK). Total RNA from WT and ALAS+/− mice was used for labeling and hybridization. RNA samples were Cy3-labeled using Agilent Low Input Quick Amp 1-color Labeling Kit (Agilent Technologies, Berkshire, UK). The level of dye incorporation was evaluated using a spectrophotometer (Nanodrop ND1000, LabTech). Labeled RNA was fragmented in the appropriate buffer (Agilent Technologies, Berkshire, UK) for 30 min at 60 °C before dilution (*v*/v) in hybridization buffer. Hybridization to 60 K high-density oligonucleotide microarray slides was performed in a microarray hybridization oven (Agilent Technologies, Berkshire, UK) overnight at 65 °C. Following hybridization, the slides were rinsed in wash buffers and immediately scanned using a DNA Microarray Scanner (Model G2505C, Agilent Technologies, UK).

Data were verified to be distributed normally. The raw data was uploaded into Agilent's GeneSpring Software, normalized and fold changes calculated. Individual gene changes are available from the corresponding author. For each group of animals the probes with an absolute >1.5-fold-change in mRNA expression between WT and ALAS+/− mice were included in subsequent analyses. These were subjected to ANOVA unequal variations test with Benjamini-Hochberg corrections. Significant 1.5-fold or more changes (*p* < 0.05) were subjected to hierarchical clustering with average linkage. The network pathways were associated using Ingenuity Pathways Analysis (Ingenuity Systems, Redwood City, CA).

## 3 Results

### 3.1 Influence of ALAS1 null allele on embryo development and survival

**Fig. 3:**
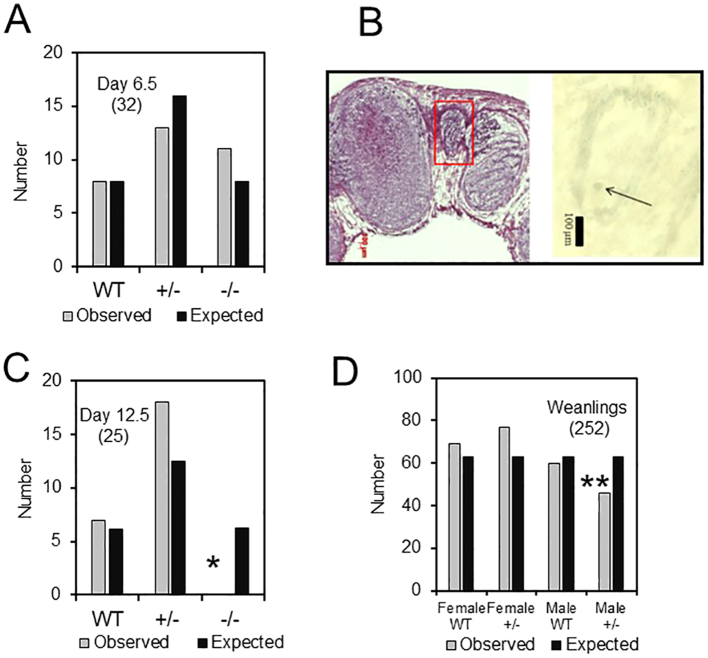
Genotypes of offspring from *ALAS1-βGEO* mice. (A) Genotypes of embryos at day 6.5 from cross of +/− x +/− mice (total embryos examined in brackets). Numbers are given in comparison with expected ratios from Mendelian inheritance (B) Section of decidua showing small embryo in decidua, probably −/−, and stained for β-galactosidase. (C) Numbers observed at day 12.5. * Absence of −/− mice and significantly different from expected by Chi squared test (*p* < 0.05). (D) Numbers of female and male +/+ and +/− weanling mice from multiple litters of +/− mice crossed with +/+ BALB/c animals. ** Significantly fewer +/− than expected; −/− mice were not observed.

The presence of WT homozygous (+/+), heterozygous null (*+/−*) or homozygous null (*−/−*) *ALAS1* genotypes were examined in embryos at days E 6.5 and at E 12.5 after an intercross of parent BALB/c *ALAS1 +/−* mice (Figs. 3A). The proportion of embryos with homozygous mutation detected at day E 6.5 was not significantly different from that expected from Mendelian inheritance confirming that expression of the *ALAS1* gene is not an absolute requirement for embryo formation [27]. Histocytochemical demonstration of β-galactosidase activity indicative of expression of the *ALAS1*-*βGEO* fusion insert expression showed some small embryos at day E 6.5 (Fig. 3B). At day E 12.5 only WT and *+/−*embryos that had continued development were detected by PCR (Fig. 3C). This demonstrated that by E 12.5 homozygous null *ALAS1* expression was lethal and indicated that the erythropoietic *ALAS2* gene which is expressed from a very early developmental stage and regulated by iron supply [5], [28], could not compensate in survival of embryos for the absence of functioning *ALAS1*. Mice could develop with only one functioning allele of *ALAS1*, either because basal expression was sufficient for requirements or by compensatory upregulation. Combined sexes of weanlings from multiple maintenance crosses between WT BALB/c and +/− mice were WT or +/− for the intact *ALAS1* allele with a ratio approximately as predicted. A small but significant decrease in the proportion of male offspring was observed. This may suggest a weak effect of *ALAS1-βGEO* dosage on early male embryonic development and survival (Fig. 3D).

5-ALA in drinking water is absorbed by adult mice and enhances hepatic porphyrin synthesis [29], [30]. However, this maternal source of 5-ALA could not rescue −/− embryos when *+/−* dams were administered 5-ALA in the drinking water for a week prior to mating with +/− males and during subsequent gestation. Only WT and *+/−* embryos were observed at day E 12.5. One explanation might be that though 5-ALA crosses the intestinal barrier it may not cross the placental barrier or was metabolized before it could do so.

Weanling BALB/c *ALAS1* +/− mice grew normally and showed no outward difference from their *ALAS1* WT (+/+) littermates. No significant difference was observed in the long-term survival between the genotypes or in motor co-ordination as assessed by the Rotarod performance test.

### 3.2 Influence of heterozygous ALAS1-βGEO on basal tissue expression

**Fig. 4:**
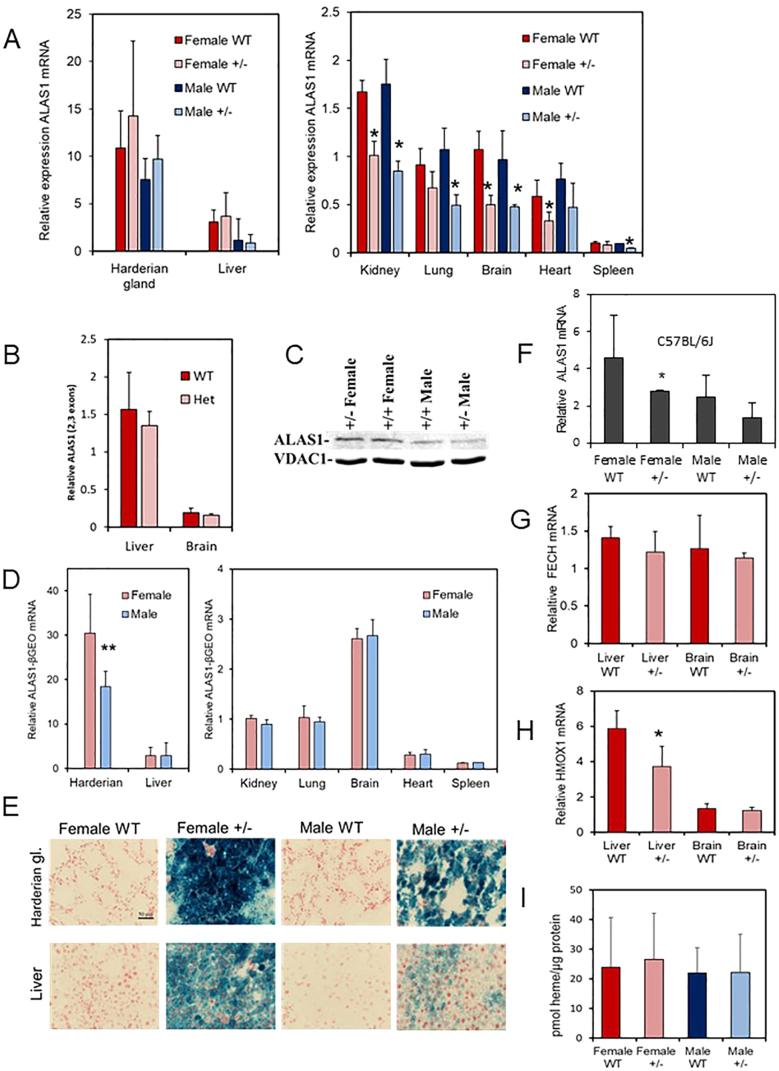
Effect of *ALAS1-βGEO* causing *ALAS1* heterozygous mice. (A) Comparison of ALAS1 mRNA levels in tissues of homozygous (WT) and heterozygous (+/−) male and female mice using PCR primers between exons 3 and 4. Relative expressions were compared arbitrarily against female +/− kidney. (B) ALAS1 mRNA in liver of WT and +/− female mice estimated using PCR primers between exons 2 and 3. (C) Western blot analysis of mitochondrial ALAS1 in female and male WT and +/− mice; mitochondrial VDAC as control. (D) Comparison of ALAS1-βGEO mRNA levels in tissues of homozygous (WT) and heterozygous (+/−) male and females (female kidney =1). (E) β-galactosidase activity in liver and harderian gland of WT and +/− mice. (F) Hepatic ALAS1 mRNA levels in *ALAS1-βGEO* C57BL/6 J mice. (G) Ferrochelatase mRNA in females. (H) HMOX1 mRNA in females. (I) Hepatic heme levels. * Significantly different from WT (*p* < 0.05, 3–10 per group). ** Significantly different from females.

The BALB/c strain was chosen as the background on which to backcross the *ALAS1-βGEO* allele, firstly, because in a screen of some inbred mouse strains BALB/c had one of the highest basal hepatic levels of ALAS1 mRNA (Supplementary Fig. 1). Secondly, because a mutation of *FECH* induces protoporphyria and liver damage in BALB/c mice but is less severe in other strains including C57BL/6 J [31]. ALAS1 mRNA levels of WT adults were much higher in the harderian gland and the liver which have higher porphyrin or heme requirements, than in a selection of other organs (Fig. 4A). Levels in harderian gland were particularly high [32]. ALAS1 expression in these two organs showed a tendency to be greater in females, perhaps indicating an association with sexually dimorphic functions, but as noted for enzyme activity levels were highly variable [33]. Levels of ALAS1 mRNA in the discrete organs kidney, lung, brain, heart and spleen were much lower than in liver and harderian gland with no evidence of sex difference.

No statistically significant influence of the *ALAS1-βGEO* fusion allele on levels of normal ALAS1 RNA in liver and harderian tissues was detected when estimated by qPCR using primers across exons 3 and 4 between which the *β*GEO construct was inserted. However, values between individuals were highly variable despite comparable ages and examination at similar times of circadian rhythm [34], although in comparison between litter mates a distinction was sometimes observed. One explanation is that rather than a theoretical level of 50% caused by disruption of one allele, limited compensatory up-regulation of the wild type allele may occur in these tissues of individual +/− BALB/c mice at time of measurement (Fig. 4A). In contrast, in the brain and those other organs examined with potentially lower constitutive demand for heme, the lower expression of ALAS1 mRNA in +/− mice in comparison to WT was mostly significant. In these organs basal expression may be less sensitive and more consistent with limited compensation for disruption of the WT allele.

Reverse transcription of liver and brain mRNA followed by qPCR using primers across exons 2–3, prior to the *β*GEO insert showed no significant difference between WT and +/− female mice suggesting no apparent distinction on initiation of transcription of the *ALAS1* and *ALAS1-βGEO* alleles (Fig. 4B). Immunoblotting of ALAS1 protein in mitochondria from young adult female liver was consistent with no marked effect in +/− mice (Fig. 4C).

Measurement by qPCR of expression of the *ALAS1-βGEO* RNA in tissues of +/− mice confirmed efficient initiation of transcription of the fusion gene which in the harderian gland was sex dependent (Fig. 4D). Although in *+/−* mice relative tissue levels of mRNA from *ALAS1* and *ALAS1-βGEO* alleles were broadly in a similar range they were not identical especially when comparing with brain. That the *ALAS1*-*βGEO* product was translated to a functional β-galactosidase enzyme was demonstrated in the liver and the harderian gland by histocytochemistry (Fig. 4E).

To check whether the ALAS1 RNA liver findings were restricted to the BALB/c strain the transgene was backcrossed for 5 generations on to the C57BL/6 J background. Although hepatic levels of ALAS1 mRNA showed no consistent effect of the +/− genotype they did appear to demonstrate more influence of the null allele than in BALB/c (Fig. 4F).

### 3.3 Expression of heme pathway enzymes in heterozygous mice

Female mice showed no evidence that the presence of the null *ALAS1-*fusion had an effect on mRNA levels of the terminal enzyme (FECH) of the heme pathway in either liver or brain (Fig. 4G). In contrast, the hepatic mRNA levels of the heme catabolism enzyme heme oxygenase 1 (HMOX1) were reduced relative to WT in +/− mice but not in the brain (Fig. 4H). These findings are compatible with a compensatory negative feedback response to maintain the size of the hepatic regulatory heme pool in unchallenged mice and hence maintain ALAS1 expression [18], [35], [36]. No difference in gross hepatic heme levels were detected (Fig. 4I) but this might not reflect any changes in the size of a pool of regulatory heme [37]. In both *ALAS1* WT and heterozygote (+/−) mice HMOX1 mRNA expression was significantly greater in the harderian gland than other tissues (data not shown) as observed in rats [32].

### 3.4 Translational control does not contribute to compensatory mechanisms in + \- mice

**Fig. 5:**
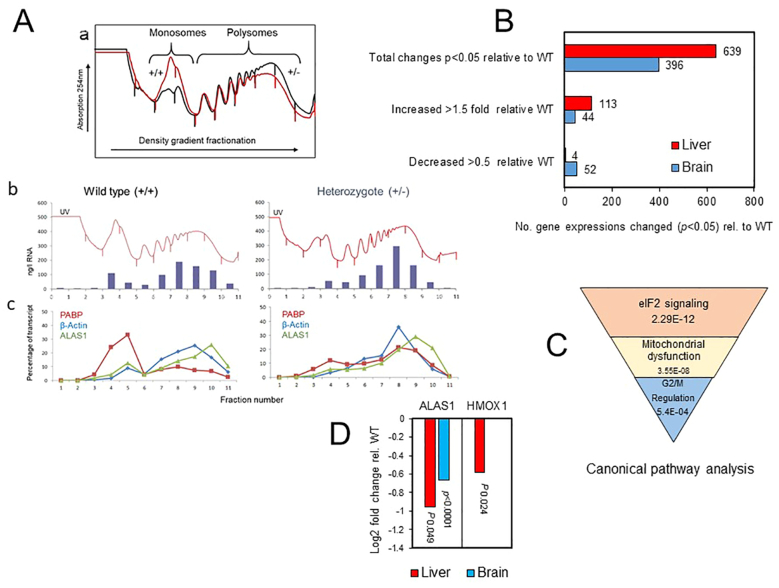
Translation and global gene expression in heterozygous ALAS1 mice. (A) Translational profiling during sucrose gradient fractionation of female WT and +/− liver RNA. (a) UV absorbance (b) RNA in fractions (c) Estimation of poly(A)-binding protein (PABP1), β-actin and ALAS1 mRNA. (B) Number of gene expression changes by transcriptomics in liver and brain from female heterozygous mice relative to WT at significance of *p* < 0.05. (C) Ranking of canonical pathway analysis of liver changes in +/− mice relative to WT. (D) Comparison of ALAS1 and HMOX1 mRNA levels and significance from WT in liver and brain as determined by transcriptomics.

ALAS1 mRNA stability in mammalian hepatocytes is one step in ALAS1 regulation [38], and may be lower in liver than in some other tissues if calculated from the ratio of ALAS1 to ALAS1-GFP RNA in a heterozygote knockout mouse [27]. Translational control is considered as at least as important for rapid gene regulation [39], [40], [41]. To investigate whether translation of the intact ALAS1 RNA from the WT allele was stimulated in heterozygous mice, hepatic RNA from WT and +/− were compared by translational profiling using sucrose density fractionation [39]. A shift in mRNA associated with 40S, 60S and 80S monosomes to polysomes indicates an increase in translation. ALAS1, β-actin and PABP1 mRNA amounts in monosomal and polysomal fractions were measured by qPCR. A modest increase in overall RNA translation was observed (Figure 5Aa) but no increase in translation of the ALAS1 RNA from the WT allele as a compensatory response for the disabled allele was detected (Figure 5Ab).

### 3.5 Comparison with global gene expression

The effects of the ALAS1-βGEO allele were also investigated by transcriptomics of mRNA levels of liver and brain from female WT and +/− BALB/c mice. A greater number of unique coding genes were up- or down-regulated (at a significance level of *p* < 0.05) in liver than brain (Fig. 5B) but in both organs degrees of change were small. Only a few of the hepatic down regulations were decreased to <50% of WT expressions. Most changes were up-regulations and consistent with increased translation of many genes as detected by translational profiling. Canonical pathway analysis computed the top two hepatic expression pathways as eIF2 signaling and mitochondrial dysfunction (Fig. 5C).

In contrast, in brain, the number of gene expressions that were down-regulated to <50% or up regulated >1.5 fold expression were similar. Only a small proportion of changes in regulation were common to both tissues (27 genes) and even fewer in the same direction (12) but these included ALAS1. Although the mRNA level of ALAS1 in liver was less in +/− than WT this was statistically only just significant (*p* 0.049) whereas the difference in brain was highly significant (*p* < 0.001) (Fig. 5D). There were no differences in expression of the other genes in the heme biosynthetic pathway (Fig. 1 *ALAD, HMBS, UROS, UROD, CPOX, PPOX and FECH*) for either tissue. In contrast, hepatic levels of HMOX1 mRNA were significantly lower in +/− than in WT genotypes (but not brain) in agreement with qPCR (Fig. 5D).

### 3.6 Response to fasting

**Fig. 6:**
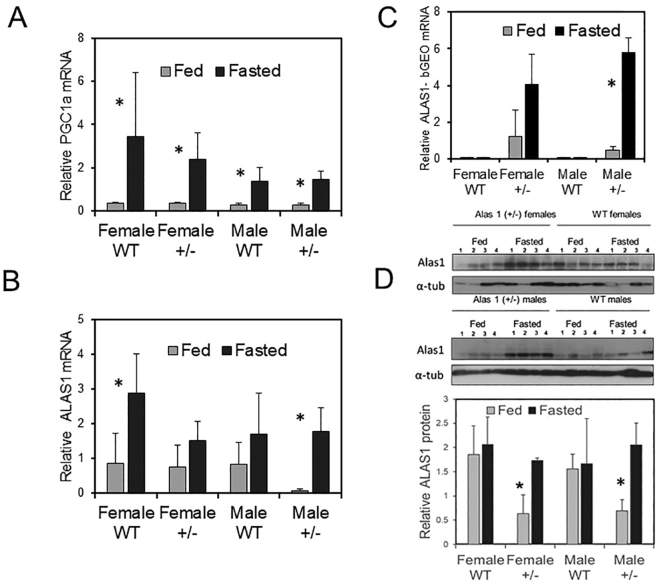
Effect of fasting on ALAS1 expression in wild type and heterozygous null mice. Diet was withheld from mice for 18 h overnight. (A) PPARGC1A expression induced particularly in females. (B) Induction of ALAS1 mRNA in males and females. (C) Induction of ALAS1-βGEO mRNA on fasting. (D) Western blotting and quantitation of total ALAS1 protein. * Significantly differently from fed mice.

In our studies, hepatic ALAS1 mRNA levels as measured by qPCR were often highly variable between individuals despite group sizes, whether mice were housed together or individually and independent of light cycles, bedding and diet suggesting sensitive responses to small changes in microenvironment or physiology. One possible source of variation was individual nutritional status. Hepatic ALAS1 expression is partially regulated by the peroxisome proliferative activated receptor, gamma, coactivator 1 alpha (*PPARGC1A* gene) expression controlling mitochondrial biogenesis, oxidative metabolism and heme demand [20]. Hepatic *PPARGC1A* and consequently *ALAS1* are upregulated by diet restriction and such a response is implicated in the precipitation of acute porphyria attacks in fasting susceptible patients [20]. Clear upregulation of hepatic *PPARGC1A* expression occurred after fasting of male and female BALB/c mice for 18 h overnight*.* This response appeared to be greater in males than females but was not significantly different between *ALAS1* WT and heterozygotes (Fig. 6A). *ALAS1* was also upregulated in both WT and +/− genotypes and there was no clear distinction with genotype despite varying levels in fed mice (Fig. 6B). Food restriction also increased expression of the *ALAS1-βGEO* product significantly in the transgenic mice (Fig. 6C).

Despite the increases in ALAS1 mRNA levels by fasting, total cellular ALAS1 protein expression rose only marginally in WT males and females. In contrast, significant increases were observed in +/− of both sexes to levels no less than WT (Fig. 6D). These findings may reflect the post-transcriptional regulatory machinery responding sensitively to heme demand [3].

### 3.7 Drug challenge

**Fig. 7:**
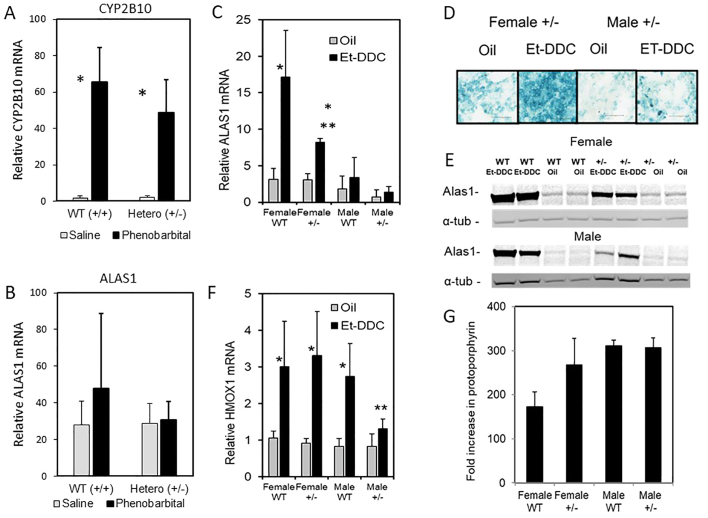
Influence of null ALAS1 allele on response to phenobarbital and 4-ethyl-DDC. (A and B) Female mice were administered a single i.p. dose of phenobarbital (80 mg/kg). CYP2B10 mRNA was markedly induced but only a non significant increase in ALAS1 mRNA was detected in WT and none in heterozygotes (+/−) after 18 h (*n* = 4). (C) WT or +/− mice were administered i.p. oil or 4-ethyl DDC (100 mg/kg) in oil and left for 18 h. ALAS1 mRNA was induced particularly in females and significantly less in +/− than WT. (D) Histochemistry showing increased β-galactosidase activity in female +/− mice. (E) Western blotting showing ALAS1 protein in WT and +/− mice. Mice were only compared in pairs but clearly show marked induction by 4-ethyl-DDC but clearly less in +/− of both sexes. (F) Induction of HMOX1 mRNA by 4-ethyl-DDC relative to oil in WT and +/− mice. (G) Fold elevation of liver protoporphyrin IX levels relative to oil vehicle in WT and +/− mice. *Significantly more than saline or oil (*p* < 0.05). ** Significantly different from WT.

Phenobarbital and some other cytochrome P450-inducing drugs will induce hepatic ALAS1 activity markedly in mice after multiple doses or prolonged administration, via an enhancer sequence distinct from the promoter region [3], [42], [43]. To explore a simple influence of phenobarbital in mice potentially deficient in ALAS1 expression, female WT and heterozygote (+/−) mice were administered a single dose of the drug. With this protocol, mRNA expression of the signature enzyme CYP2B10 was increased markedly after 18 h in both genotypes (Fig. 7A). However, at this time of measurement only marginal induction of ALAS1 mRNA was detected by qPCR in WT and not at all in +/− mice (Fig. 7B) suggesting that for this degree of P450 increase only minimal elevation of transcription was required.

The agent 4-ethyl-DDC is one of a group of drugs that during their hepatic metabolism by some CYP450s the hemoprotein becomes inactivated by N-alkylation of the heme moiety in a suicidal mechanism and N-ethyl-protoporphyrin IX is released [3], [23]. The N-alkyl porphyrin acts as an inhibitor of ferrochelatase leading to marked accumulation of the substrate protoporphyrin IX. The resulting decrease in heme formation compounded by drug-induced induction of HMOX1 via antioxidant response, causes continual feedback up-regulation of ALAS1 activity [3], [26]. Both WT and +/− genotypes showed marked induction of hepatic ALAS1 mRNA and protein compared to controls 18 h following administration of a single dose of 4-ethyl-DDC to females, but much less in males. However, this induction was markedly less in +/− animals than WT (Fig. 7C and D). Histocytochemistry of livers from +/− mice illustrated the upregulation of *ALAS1-βGEO*, particularly in females (Fig. 7E). Induction of HMOX1 mRNA following administration of 4-ethyl-DDC occurred in both sexes and was affected by *ALAS1* genotype in males but not significantly in females (Fig. 7F). Because of the temporal and other regulatory factors the levels of accumulated hepatic protoporphyrin caused by these drugs are not a simple reflection of ALAS1 activity [26]. Indeed, although 4-ethyl-DDC caused massive accumulation of protoporphyrin IX in the liver of all mice this was little affected by reduced gene dosage of intact *ALAS1* at the time of measurement (Fig. 7G).

## 4 Discussion

In many studies of genetically modified mice, only null individuals are investigated if the gene is not essential for life, e.g. in some aspects of cell signaling in which null gene mice survive. Heterozygous mice are studied less often but are important as penetrance of the intact allele may be more complex than the theoretical gene dosage of 50% of homozygous mice due to potential compensatory mechanisms. The expression of *ALAS1* is essential to many cellular systems in mammalian biology including its central role in mitochondria, microsomal cytochrome activities and in aspects of intra- and inter-cell signaling. Consequently, it is not surprising that embryonic −/− mice did not survive past the first few days after which they have to develop mitochondrial respiration independent from maternal tissues [27]. There was no major effect of heterozygosity on numbers of mice at weanling or long-term survival although there was a statistically significant small decrease in the proportion of males born, of unknown biological relevance. Clearly most developing and adult mice were able apparently to cope with only a half intact gene dosage under unchallenged conditions. In another *ALAS1* knockout model, aged mice show some symptoms of impaired glucose tolerance and insulin resistance with evidence of skeletal muscle mitochondrial attenuation [19], [37]. These aspects were not studied in the present investigations but we did find preliminary evidence of changes in the function of hepatic mitochondrial complexes in older animals (Supplementary Fig. 2).

The regulation of hepatic ALAS1 expression is sensitive and complex not only at a transcriptional level but also in RNA and protein processing as well as in mitochondrial import and turnover stages which is not surprising for such a critical gene in cellular function. These posttranslational actions might compensate for any deficiency in the availability of the active enzyme. In our model the basal hepatic expression of ALAS1 RNA in individual unchallenged mice sometimes showed effects of heterozygosity but these could be highly variable and inconsistent despite attempts to standardize conditions, ages and timing. Such variability was also seen in the harderian gland. Both tissues seem to respond sensitively to demand of the heme synthesis pathway and with different regulatory influences than in other tissues [3], [32]. In contrast, in those other organs that were examined ALAS1 RNA levels were much more consistent with levels approximately 50% of WT. In the previously published *ALAS1* knockout model, heterozygosity for the null allele on a mixed genetic background of older mice was reported as approximately 50% of the ALAS1 message compared to the WT for liver, muscle and fat [19]. This could suggest that in BALB/c ALAS1 +/− mice the liver under basal circumstances might have enough reserve capacity or an ability to compensate by a variety of mechanisms for depleted gene dosage. It may be pertinent that inbred mouse strains can vary in hepatic levels of *ALAS1* RNA as measured by qPCR. This can be explained partially by an inhibitory B2 SINE retrotransposon in the *ALAS1* promoter region of some strains, but with unknown physiological significance [12].

Another potentially complicating factor in the hepatic expression of *ALAS1* compared to some other tissues is the complexity of cellular and nuclear ploidy. Hepatocytes from both humans and mice can be binucleate as well as mononucleate and are predominantly tetraploid or greater. This may shift markedly in either direction under some physiological and pathological conditions including iron status [44]. The influence of different ploidy states in cells, thus variable gene copy numbers, on selected gene expressions in individual hepatocytes and contributions to expression of total cell populations in the liver following physiological, pharmacological or chemical stimulation in genetically modified animals, are far from understood and often ignored [45], [46].

Hepatic ALAS1 activity is increased by fasting mediated through induction of *PPARGC1A* expression [20], [21]. In our studies, PPARGC1A, ALAS1 and ALAS1-βGEO mRNAs were all induced by fasting but there were no clear, consistent effects of the null allele. Curiously, in these experiments total cellular ALAS1 protein in +/− was significantly lower than WT in fed animals, but on fasting was increased to levels in WT that despite induced mRNA did not manifest in higher protein levels. As before, there was considerable inter-individual variability, but this could illustrate the considerable feedback control in the hepatic ALAS1 expression to maintain heme and mitochondrial homeostasis under physiologically normal conditions. Unlike basal states, many drugs induce ALAS1 predominantly via CAR and PXR binding on an enhancer sequence upstream of the *ALAS1* coding gene and separate from the promoter region responsive to cellular and physiological variability [42]. With the porphyrogenic drug 4-ethyl-DDC the influence of a null allele on ALAS1 expression was clear. Both mRNA and protein showed marked reduction in +/− mice. However, at the time of termination there was no marked effect on the degree of hepatic protoporphyria observed.

The decreased hepatic HMOX1 mRNA expression in unchallenged +/− mice is compatible with the concept of feedback regulation, via a regulatory heme pool, as one compensation for a decline in heme availability due to loss of a functioning *ALAS1* allele [3]. However, HMOX1 mRNA expression was induced to a similar degree in both WT and +/− female mice after treatment with 4-ethyl-DDC despite the fact that inhibition of ferrochelatase and heme synthesis would have occurred due to N-alkylated protoporphyrin formed from cytochrome P450 [26]. The role of HMOX1 and consequences following induction with drugs is complex [3]. In fact, there is good evidence that the mode of action of HMOX1is not only to degrade heme but has other non enzymic roles in stress response [47]. In contrast to the liver, down regulation of HMOX1 was not observed in the brain of +/− mice untreated mice. One explanation might be that in the brain, the ALAS1-HMOX1 axis is not as sensitive to heme supply as in the liver. Both ALAS1 and HMOX1 mRNA induction have been observed in cultured primary cortical neurons undergoing senescence [48].

The main reason for peripheral neurological symptoms in human AIP seems to be the neurotoxicity of plasma 5-ALA produced by the induction of hepatic ALAS1 rather than heme deficiency [2], [9], [49]. Indeed a promising treatment against neurovisceral attacks is the use of a siRNA targeting hepatic ALAS1 mRNA [43], [50] However, in a mouse model of AIP involving phenobarbital administration to a HMBS −/− mutant, modulation in mitochondrial complexes consistent with heme deficiency have been reported for brain, liver and skeletal muscle [51], [52]. The observation that brain displayed more clear effects of one intact *ALAS1* allele than the liver suggests the possibility that the effect of semi ALAS1 dosage might have subtle influences on neuronal heme supply in development and in responding to stress and disease [53], [54], [55]. This might support a view that heme deficiency may play a role not yet recognized clinically in some diseases but not necessarily in recognized porphyria disorders.

It is interesting to speculate why a gene so inducible in the liver and sensitive to changes in heme demand and restrictions as in porphyrias shows no marked indication for compensatory up-regulation of the WT allele in heterozygotes after a strong drug stimulatory signal. Perhaps the demonstrable expression of the *ALAS1-*β*GEO* product may mean that the cell does not recognize a deficiency in expression of the WT *ALAS1* copy in this scenario. Some studies suggest that expression of different gene alleles is purely on a stochastic basis and thus might not easily adapt to compensatory expression in this model [56]. Compensation may also vary on whether changes in gene dosage are caused by a mutational knockout or by knockdown [57]. Further detailed in vivo or in vitro studies of silencing or producing inactive variants of one *ALAS1* allele could address these questions.

The present studies demonstrate the potential complexity of expression of *ALAS1* in different tissues and circumstances in the presence of a single intact ALAS1 allele. This may explain partly why human disorders associated with a phenotypic variant have not been reported.

## Authorship contributions

Participated in research design: Chernova and Smith.

Conducted experimental work: Vagany, Robinson, Chernova.

Wrote or contributed to the writing of the manuscript: Chernova and Smith

## Appendix A Supplementary data

Supplementary material. Image 1
