# Supplementary material for: Complex response to physiological and drug-induced hepatic heme demand in monoallelic ALAS1 mice
Source: Mol Genet Metab Rep. 2021 Nov 12;29:100818. doi: 10.1016/j.ymgmr.2021.100818 (PMC8639769; doi:10.1016/j.ymgmr.2021.100818)
Supplement: Supplementary file 1 — Supplementary material. [file mmc1.pdf]

Vagany et al. *Complex response to physiological and drug-induced hepatic heme demand in monoallelic ALAS1 mice*

Supplemental material.

One Table and two Figures.

Gene mRNA	Direction	Primers
ALAS1 e3	F	TCTTCCGCAAGGCCAGTCT
	R	TGGGCTTGAGCAGCCTCTT
ALAS1 e2	F	ACCCTGTCCACATCAGCTG
	R	GCCTTGGCAGTTTTCTCTTTC
ALAS1β-GEO	R	GTTTTCGGGACCTGGGACTT
FECH	F	CATGCCAAGACCACCAAAC
	R	TTAACATCAATATGCCCGTTTTT
HMOX1	F	CACTTCGTCAGAGGCCTGCTA
	R	GTCTGGGATGAGCTAGTGCTGAT
β-ACTIN	F	GATTACTGCTCTGGCTCCTAGCA
	R	GTGGACAGTGAGGCCAGGAT
β-MICROGLOBULIN	F	CACTTCGTCAGAGGCCTGCTA
	R	GTCTGGGATGAGCTAGTGCTGAT
PGC1A	F	ATGTGTCGCCTTCTTGCTCT
	R	CACGACCTGTGTCGAGAAAA
CYP2B10	F	CAATGTTTAGTGGAGGAACTGCG
	R	CACTGGAAGAGGAACGTGGG
PABPC1	F	CCTCCTTCAGGTTACTTCATGGC
	R	GGACTTGGTCTTAGTTGAGCAAT

Supplementary Table 1. PCR primers used for expression estimation. Gene abbreviations shown in upper case. F for forward primer R for reverse.

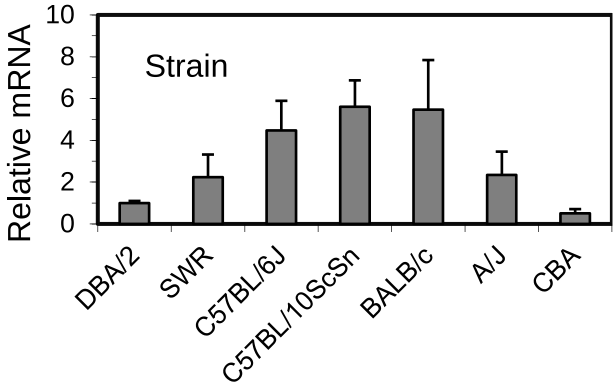

Supplementary Figure 1. Comparison of levels of ALAS1 mRNA in livers of strains of male mice determined by qPCR as described previously (Chernova et al., 2008).

Chernova T, Higginson FM, Davies R and Smith AG (2008) B2 SINE retrotransposon causes polymorphic expression of mouse 5-aminolevulinic acid synthase 1 gene. *Biochem Biophys Res Commun* 377 (2): 515-520.

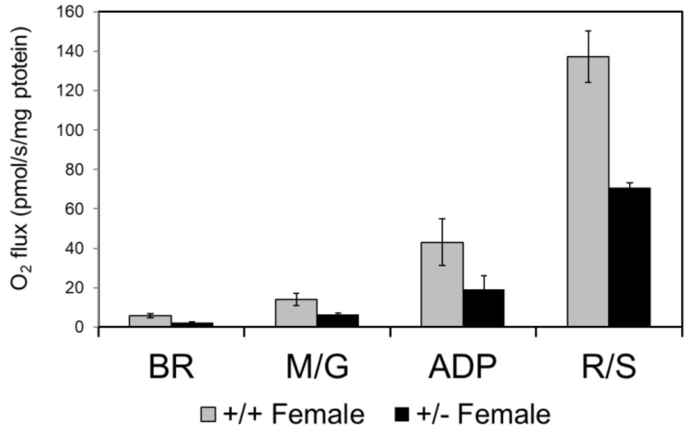

Supplementary Figure 2. Example of oxygen consumption in the liver of female ALAS1 +/+ and +/- mice litter mates. Mitochondrial oxygen consumption with an Oroboros two chamber titration-injection oxygraph (Oroboros Oxygraph2-k) by the liver from 20 week wild type (+/+) and heterozygous (+/-) female mice. The basal respiration (BR) rates were assayed in the absence of exogenous respiration substrates. For coupled (state 3) assays, complex I activity was assayed in the presence of 2mM malate (M), 10mM glutamate (G) and 5mM ADP. Complex II was assayed in respiration buffer supplemented with 1mM rotenone (R), 10mM succinate and 5mM ADP. Basal, Complex I and Complex II-driven respiration were reduced in ALAS1 +/- mice compared to wild type (ALAS1 +/+). Not all comparisons of mice showed this effect in a similar variation as observed with ALAS1 mRNA levels in younger adults. Heme deficiency has been reported to influence mitochondrial complexes (Atamna et al., 2001). We thank members of the Dr L M Martins laboratory (MRC-Toxicology Unit) for advice.

Atamna H, Liu J and Ames BN (2001) Heme deficiency selectively interrupts assembly of mitochondrial complex IV in human fibroblasts: relevance to aging. *J Biol Chem* 276 (51): 48410-48416.
